# No clear evidence for an innate left-to-right mental number line

**DOI:** 10.1073/pnas.2306099120

**Published:** 2023-07-03

**Authors:** Benjamin Pitt, Daniel Casasanto, Steven T. Piantadosi

**Affiliations:** ^a^Department of Psychology, University of California, Berkeley, CA 94720; ^b^Department of Psychology, Cornell University, Ithaca, NY 14853

In their paper, Giurfa, Marcout, Hilpert, Thevenot, & Rugani (1) seek to clarify the origins of spatial–numerical associations in a study of honeybees. Like studies of other animals and human neonates (2–4), this study shows a robust behavioral asymmetry: rightward movement in response to more numerous dot arrays and leftward movement in response to less numerous arrays. These results are provocative, but there are reasons to doubt that humans or animals have an innate left-to-right mental number line.

First, the observed behavioral asymmetries can be explained without appeal to any space–number associations. Sensory information is often processed differently by each side of the nervous system, causing behavioral asymmetries in a variety of animals, including invertebrates (5–7). For example, in many species, stimuli with low spatial frequency are best viewed in the left visual hemifield and stimuli with high spatial frequency are best viewed in the right hemifield. Since spatial frequency is confounded with numerosity in dot arrays, this lateralization should cause animals to turn in different directions in response to different numbers of dots, on the basis of spatial frequency alone (6, 7). Animals also respond asymmetrically on the basis of emotional motivation, habitually performing approach actions on the right side and avoidance actions on the left (3). If they learn to associate dots with reward (and more dots with more reward), then the number of dots displayed should indirectly influence animals’ turning direction, even in the absence of a mental number line (3). These asymmetries in neurological function may explain the observed effects and, importantly, they are not akin to space–number associations, as they do not reflect representations of space or number. Indeed, it is difficult to imagine the selective pressures that might yield a direction-specific mental number line; in the wild, animals do not encounter more food, predators, or conspecifics on one side of their body than the other.

Second, even if we assumed that the observed behavioral asymmetry reflected a mental number line, there is no evidence that it has left-to-right direction. If smaller numbers are mentally to the left of larger numbers, a bee searching for a sugar reward at three should go to the *left* when it finds a number larger than three, and to the *right* when it finds a number smaller than three. Yet, the bees in this study (like animals in previous studies) show the opposite pattern, consistent with a right-to-left number line.

Finally, many studies in humans suggest that the direction of the mental number line is determined by cultural experience, not biological defaults. Space–number associations vary across cultures and flexibly change with just a few minutes of training (8). Populations with little exposure to symbolic number—like young children and indigenous adults—show no sign of a left-to-right mental number line, organizing dot arrays equally in each direction (9, 10). Taken together, these findings suggest that what appears to be a direction-specific mental number line shared across species may actually be “mere resemblances reflecting completely different underlying mechanisms” (3).
